# Diagnosis of childhood and adolescent growth hormone deficiency using transcriptomic data

**DOI:** 10.3389/fendo.2023.1026187

**Published:** 2023-02-14

**Authors:** Terence Garner, Ivan Wangsaputra, Andrew Whatmore, Peter Ellis Clayton, Adam Stevens, Philip George Murray

**Affiliations:** ^1^ Division of Developmental Biology and Medicine, Faculty of Biology, Medicine and Health, University of Manchester and Manchester Academic Health Science Centre, Manchester, United Kingdom; ^2^ Department of Paediatric Endocrinology, Royal Manchester Children’s Hospital, Manchester, United Kingdom

**Keywords:** growth hormone deficiency, transcriptome (RNA-seq), machine learning, growth hormone, random forest - ensemble classifier

## Abstract

**Background:**

Gene expression (GE) data have shown promise as a novel tool to aid in the diagnosis of childhood growth hormone deficiency (GHD) when comparing GHD children to normal children. The aim of this study was to assess the utility of GE data in the diagnosis of GHD in childhood and adolescence using non-GHD short stature children as a control group.

**Methods:**

GE data was obtained from patients undergoing growth hormone stimulation testing. Data were taken for the 271 genes whose expression was utilized in our previous study. The synthetic minority oversampling technique was used to balance the dataset and a random forest algorithm applied to predict GHD status.

**Results:**

24 patients were recruited to the study and eight subsequently diagnosed with GHD. There were no significant differences in gender, age, auxology (height SDS, weight SDS, BMI SDS) or biochemistry (IGF-I SDS, IGFBP-3 SDS) between the GHD and non-GHD subjects. A random forest algorithm gave an AUC of 0.97 (95% CI 0.93 – 1.0) for the diagnosis of GHD.

**Conclusion:**

This study demonstrates highly accurate diagnosis of childhood GHD using a combination of GE data and random forest analysis.

## Introduction

Growth hormone deficiency (GHD) is a rare but important cause of short stature with a prevalence of approximately 1 in 4000 (1). Consensus guidelines recommend an approach integrating auxological, biochemical and radiological data for the diagnosis of GHD in childhood and adolescence (2). Pharmacological stimulation tests, in which a cut-off level is selected for peak growth hormone (GH) levels below which the child is diagnosed with GHD, remain key to the diagnosis of GHD despite many known problems with these tests. These problems include poor reproducibility (3) and that the peak GH level achieved is affected by the pharmacological stimulus used (4), the GH assay (5) and body composition (6, 7). In one survey the peak GH level utilized for diagnosis varied between 6 and 10 μg/L in nine European and US national guidelines (8).

In addition to these problems most of the pharmacological stimulation tests require fasting and all require multiple blood samples. Both of these requirements can be challenging for small children, particularly those with significant medical problems e.g. history of extreme prematurity where venous access may be very challenging, in children with needle phobia or autistic spectrum disorders. Adverse effects such as vomiting and nausea are common and rarely serious adverse events such as cerebral edema have been associated with these tests (9). We have therefore sought to develop a gene expression-based test as a potential replacement for pharmacological stimulation tests. This would require only a single blood sample and, as no pharmacological stimulant would be needed, would avoid any significant adverse effects.

Gene expression based analysis has been utilized in the diagnosis of interstitial lung disease (10), kidney disease (11), atrial fibrillation (12), autism (13) and in the prognosis and classification of tumors (14–16). For both autism and atrial fibrillation peripheral blood gene expression was used for diagnosis (12, 13). We therefore hypothesized that gene expression signatures in peripheral blood could be a diagnostic tool for GHD with the potential to replace pharmacological stimulation tests. In an initial study (17) we compared gene expression profiles between 98 children with GHD enrolled in the PREDICT study (18) and 26 healthy control children whose gene expression data were obtained from online datasets. After selecting the 271 probesets whose expression correlated with peak GH levels a Random Forest classifier gave an Area under the Receiver Operating Characteristic Curve (AUC-ROC) of 0.95 (sensitivity 96%, specificity 100%) for predicting the diagnosis of GH indicating this had the potential to be an excellent diagnostic test (17). There were, however, several limitations to that initial study: 1) the control subjects used were healthy rather than short stature controls 2) the patient and control children were assembled from different studies and 3) GH stimulation test and assay were not standardized in the PREDICT study.

The aim of this study was to assess the utility of gene expression data for the diagnosis of GHD in a prospectively recruited cohort of children and adolescents undergoing pharmacological stimulation tests at a single tertiary pediatric endocrinology center.

## Methods

### Ethics and patients

This study was approved by the Bradford Leeds Research Ethics Committee (Reference 18/YH/0226 IRAS ID 231325) and conducted in accordance with Good Clinical Practice and the Declaration of Helsinki. Informed consent was obtained either from parents or from the young person themselves where they were over 16 and had capacity to give consent.

Patients were recruited from clinics of the Paediatric Endocrinology Department at the Royal Manchester Children’s Hospital. Every patient attending the outpatient medical investigation unit for either an arginine or glucagon stimulation test was invited to take part in the study between 1^st^ November 2018 and 31^st^ January 2019. 44 patients were invited to take part and 27 (59%) both attended for their appointment and agreed to take part in the study. For three patients a result for the pharmacological stimulation test was not available due to difficulties with obtaining venous blood leaving 24 patients in the study. Auxological, biochemical and radiological data were obtained from the patients records. SDS scores for auxological data were calculated with Auxology 1.0 (Pfizer, New York, USA) using UK 1990 Cole Reference data.

### Stimulation test protocols, sex steroid priming and biochemical assays

Arginine and glucagon tests were the only GH stimulation tests used in our institution during the study period. Protocols for the arginine and glucagon stimulation tests used in our institution are available at https://mft.nhs.uk/app/uploads/2022/03/Paediatric-DFT-Protocols-V7_Feb-2022.pdf (accessed 22nd August 2022). Sex steroid priming is given in our unit for pre-pubertal girls >8 years or boys >9 years undergoing stimulation tests. Ethinylestradiol 10-20 micrograms is given once daily for 3 days prior to the test for both boys and girls. A normal test result in our institution is indicated by a peak GH level ≥ 7 μg/L. GH, IGF-I and IGFBP-3 were measured on the IDS iSYS assay (Immunodiagnostic systems, Tyne and Wear, UK). The GH assay used is standardized to the recombinant GH calibration standard World Health Organization 98/574 and complies with recommendations on assay standardization (19).

### Statistics, regression and random forest analysis

Differences in demographic characteristics were assessed *via* a Mann Whitney U test or Fisher’s Exact test. A Random Forest algorithm (20) was used to predict GHD status. The data were unbalanced (8 GHD subjects and 16 controls) and with an unbalanced dataset Random Forest poorly predicts the minority class (in this case GHD subjects). To overcome this problem a synthetic minority over-sampling technique (21) was used to rebalance the dataset prior to Random Forest prediction using age, gender and transcriptomic data. The predictions were assessed based on the AUC-ROC and the out-of-box ROC curve (OOB-ROC) as a validation set. Identifying those probesets most likely to contain predictive capacity was achieved with the use of Boruta (22). All statistical analyses were performed using R 4.0.0.

Random Forest analysis does not require separate test and validation data sets as the OOB-ROC functions as a validation data set. In developing the random forest algorithm hundreds or thousands of decision trees (in our case 1000 trees) are created. Each tree is generated using a random selection of input variables and randomly selected two thirds of the subjects. Each tree produced a classification vote and the majority vote across all trees determined final classification. For each tree there was a random one third of subjects whose data was not used in generating that tree – these data were then used to generate the OOB-ROC which essentially functions as a validation data set.

Boruta is used to define which of the input variables for the Random Forest are contributing to the predictive power. It does this by permuting the data (randomly shuffling the variables – in this case gene expression levels) to break any link between the input variables and outcome measured. These permuted variables are referred to as “shadow” variables and Boruta then runs a Random Forest algorithm using the shadow variables (with the same outcome measure as the original dataset) to define the range of predictive power of these shadow variables. The predictive power of the variables in the original data are then compared to the range of predictive power shown by the shadow variables. On the basis of that comparison the original variables (in this case individual gene expression levels) are classified as confirmed, tentative or rejected.

### Gene expression analysis

Peripheral blood samples (2.5 ml) were taken into PAXgene tubes (Qiagen, Manchester, UK) and stored at -80°C for the separation of total RNA as a single batch. Total RNA was submitted to the Genomic Technologies Core Facility. Quality and integrity of the RNA samples were assessed using a 4200 TapeStation (Agilent Technologies) and then libraries generated using the TruSeq^®^ Stranded mRNA assay (Illumina, Inc.) according to the manufacturer’s protocol. Briefly, total RNA (1ug) was used as input material from which polyadenylated mRNA was purified using poly-T, oligo-attached, magnetic beads. The mRNA was then fragmented using divalent cations under elevated temperature and then reverse transcribed into first strand cDNA using random primers. Second strand cDNA was then synthesized using DNA Polymerase I and RNase H. Following a single ‘A’ base addition, adapters were ligated to the cDNA fragments, and the products then purified and enriched by PCR to create the final cDNA library. Adapter indices were used to multiplex libraries, which were pooled prior to cluster generation using a cBot instrument. The loaded flow-cell was then paired-end sequenced (76 + 76 cycles, plus indices) on an Illumina HiSeq4000 instrument. Finally, the output data was demultiplexed (allowing one mismatch) and BCL-to-Fastq conversion performed using Illumina’s bcl2fastq software, version 2.17.1.14. BAM files were used to generate raw counts were mapped to the human reference genome (GCA_000001405.15 GRCh38 from NCBI).

The EdgeR package for R 4.0.0 (23, 24) was used to assess gene expression. Genes were filtered to remove low expression features. Across all cells, genes with fewer than 15 total counts were removed; the minimum count per cell for a feature to be considered expressed is 10. The large group size was set at 10 samples and genes had to be expressed in 70% of cells of smaller groups to pass filtering. Gene level normalization was performed using TMM (trimmed mean of M values). A block design in EdgeR was used to control for the impact of confounding factors (age and gender).

Gene ontology was performed using biological process data in the WebGestalt online toolkit (25). Clusters of related ontologies were defined and primary pathways identified using weighted set cover (26).

Sequence data is available *via* NCBI Gene Expression Omnibus as GSE190502. Individual patient data (age, gender, type of GH simulation test, peak GH level and use of sex steroid priming) is included in GSE190502 and as **Supplementary Table 1**.

## Results

### Patients

Of the 24 patients who were enrolled in the study and completed the diagnostic test eight were diagnosed with GHD and 16 did not have GHD. Of the eight patients with GHD five were diagnosed with GHD with a single test due to the presence of MRI abnormalities of the hypothalamo-pituitary axis. Two patients had isolated anterior pituitary hypoplasia, one anterior pituitary hypoplasia with an arachnoid cyst, one anterior pituitary hypoplasia combined with a thin stalk and multiple sclerosis and one patient had a small anterior pituitary with bulky optic nerves (this patient has a diagnosis of neurofibromatosis type 1). Two patients had a normal pituitary exam and were diagnosed with GHD on the basis of two independent GH stimulation tests. One patient was born small for gestational age, had a normal MRI pituitary and a CHARGE syndrome phenotype and was started on GH after a single test. Three patients in the non-GHD group had genetic disorders – one with Prader-Willi syndrome, one with Wiedemann-Steiner syndrome, one with a chromosome 21q deletion and one patient had a peroxisomal disorder. In the GHD group one patient had a genetic condition – neurofibromatosis type 1.

Six patients had glucagon stimulation tests and 18 arginine stimulation tests. All patients undergoing glucagon stimulation testing had a previous failed arginine stimulation test. The test was primed with sex steroids in seven cases. There were 13 male and 11 female patients. 18 patients were prepubertal, 2 patients had just started puberty (one girl at breast stage 2 and one boy with testicular volumes of 5 mL bilaterally) and 4 patients had either completed puberty or were near the end of puberty. Baseline clinical data (auxology and biochemistry) are given in **Table 1**. There was no significant difference between the GHD and non-GHD group for age, gender, birth weight SDS, height SDS, parental height adjusted height SDS, growth velocity SDS, weight SDS, BMI SDS, bone age delay, IGF-I SDS or IGFBP-3 SDS.

**Table 1:** Baseline characteristics of study participants.

	GHD (n=8)	GH sufficient patients (n=16)	P-value
Age (years)	8.6 (9.0)	9.0 (5.8)	0.52
Male Gender (n, %)	6 (75)	7 (43)	0.21
Birth Weight SDS	-0.7 (0.7)	-1.1 (1.7)	0.51
Height SDS	-3.0 (1.0)	-2.5 (0.7)	0.48
Weight SDS	-2.5 (2.4)	-2.1 (1.5)	0.98
Body Mass Index SDS	0.3 (1.7)	-0.1 (1.4)	0.32
Parental Adjusted Height SDS	-2.1 (0.3)	-2.5 (1.7)	0.70
Growth Velocity SDS	-1.5 (1.7)	-1 (3.3)	0.52
Prepubertal (N, %)	7 (87)	11 (68)	0.62
Bone Age Delay (years)	1.5 (1)	1.7 (1.4)	1.0
IGF-I SDS	-2.2 (1.7)	-1.5 (1.5)	0.20
IGFBP-3 SDS	-1.4 (1.6)	-0.8 (1.9)	0.33

Data is given as median (interquartile range) unless otherwise stated. Patients who had reached end of growth are not included in the analysis of growth velocity or analysis of height SDS.

Our study included 3 patients who were evaluated as part of an end of growth assessment (having previously been treated with recombinant human GH for GHD). These subjects were not GHD with peak GH levels of 9.1 μg/L, 9.5 μg/L and 31 μg/L to arginine stimulation testing. End of growth patients previously treated with recombinant human GH were not included in the analysis of growth velocity SDS and or height SDS. The three patients evaluated as part of an end of growth assessment had stopped treatment with recombinant human growth hormone a minimum of six weeks before the end of the test, all had a low likelihood of continuing GHD and were thus retested as per recommendations in international guidelines (27). All other patients had not received recombinant human growth hormone treatment at the time of the study.

### Gene expression data

Of the 271 genes previously identified as predictive of GHD in relation to normal controls (17) 208 could be annotated to an ensemble external gene name and 160/208 passed through low expression filtering in the dataset of GHD patients (n=8) and short stature controls (n=16). A strict threshold to define expression was used in our new dataset hence the reduction in number of genes expressed in both datasets. Transcriptomic data was Log_2_ normalized (TMM) and age and gender were treated as confounding factors in the data. Seven out of the 160 genes in the predictive group had significant differential expression (0.02<p<0.05 - *NOTCH3*, *LAYN*, *SHF*, *GRB10*, *SH3GLB2*, *CYB5A* and *SGSM2*).

**Figure 1:**
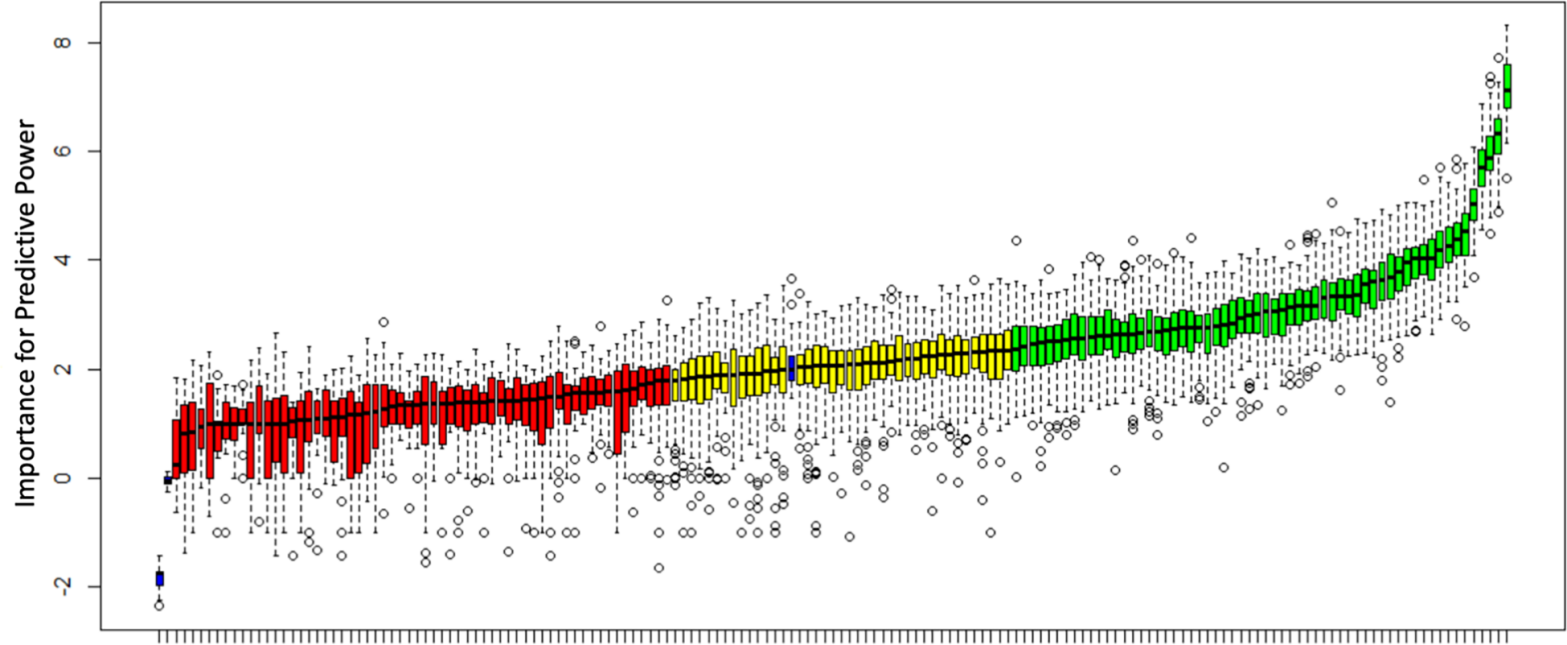
Analysis of predictive power for the diagnosis of GHD using Boruta. All 160 genes utilized in this study are shown and predictive power compared to shadow variables (blue bars) generated from permuted data. Genes are classified as confirmed, tentative or rejected based on a comparison with the shadow variables. 60 genes were confirmed (green), 40 tentative (yellow) and 60 rejected (red). A median importance score ≥ 2.359 resulted in a confirmed status, a score ≥1.785 and < 2.359 resulted in tentative status and a score <1.785 rejected status.

A random forest algorithm after adjusting for imbalanced numbers using SMOTE (final data used 44 subjects: 20 short stature controls and 24 GHD patients) in this cohort gave an AUC of 0.95 (95% CI 0.89 – 1.00) for the diagnosis of GHD. Boruta (using 99 iterations) was able to identify 100/160 genes with predictive capacity greater than permuted data within the dataset (60 confirmed and 40 tentative, see **Figure 1** and **Supplementary Table 2**).

**Figure 2:**
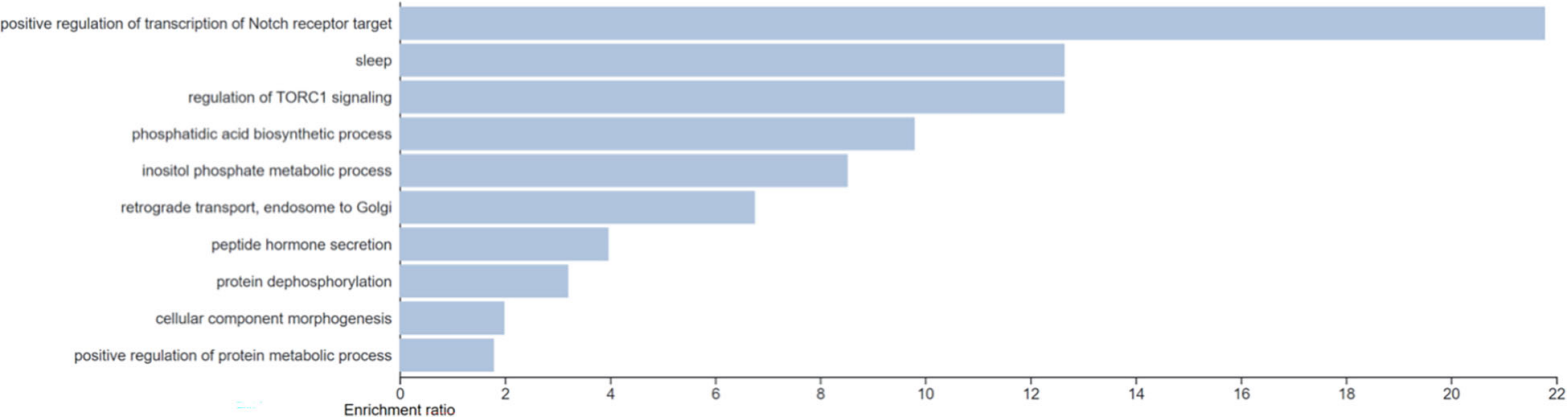
Gene ontology analysis of the genes with predictive capacity with clustering of the top 50 biological pathways *via* weighted set cover. 10 clusters of gene ontology were identified (0.0037 < p < 0.018).

Gene ontology of the genes with predictive ability identified a range of associated biological processes. The top fifty biological processes clustered by weighted set cover to 10 clusters of ontologies (see **Figure 2**) 0.0037<p<0.018. Pathways identified included regulation of TORC1 signaling and inositol phosphate metabolic processes.

We had previously identified the 10 genes with greatest predictive value in classification of GHD in relation to normal controls when combined with genetic data (17). Of these two were validated as being of predictive value in classification of GHD in comparison to short stature controls (*NRXN1 & PTGDS*).

## Discussion

Our previous study (17) highlighted the potential for GE data to be used for the diagnosis of childhood GHD with a very promising AUC for predicting GHD status of 0.95 (95% CI 0.91 – 0.99) and our current study confirms that potential with an AUC of 0.97 (95% CI 0.93 – 1.0). We have changed from using Affymetrix HU 133 plus 2.0 arrays to an RNA sequencing based approach to measure GE thus moving from measurement of relative to absolute concentrations of RNA. This change in technique did mean that we could not use exactly the same random forest algorithm in this study as was generated in our original study, however, we did utilize the same set of 271 genes whose expression was related to peak GH levels in the original study. Of the 271 genes utilized in our original study 160 were present in the RNAseq data. The reduction in number is likely to reflect the strict cut-off used for filtering low expression genes in this study. We believe an RNA sequencing based approach is likely to achieve more widespread uptake in the future as many labs already use next generation sequencing approaches for DNA studies. The AUC for the gene expression based test is better than the current baseline tests used in the diagnosis of GHD namely IGF-I with an AUC of 0.73 and IGFBP-3 with an AUC of 0.8 (28).

The major limitation of our previous study was that we compared GE data from children with GHD to healthy controls (likely to be of normal stature) accumulated from several different datasets. In this study the control cohort was short non-GHD children who had undergone GH stimulation testing. One significant strength of the study is that we recruited an unselected real-world sample of patients undergoing GH stimulation tests who are likely to be generally representative of the patients that pediatric endocrinologists select for testing. There were no significant differences between the short non-GHD and GHD children for age, gender, height SDS, parental adjusted height SDS, growth velocity SDS or BMI SDS. The absence of any significant difference in height or parental adjusted height SDS between the GHD and non-GHD short stature children may be due to the low numbers of GHD subjects. IGF-I and IGFBP-3 SDS concentrations were lower in the GHD group but this was not significant. In children with GHD IGF-I and IGFBP-3 concentrations can be in the low or low normal range with suggested cut-offs for the diagnosis of childhood GHD of -1.6 SD for IGF-I and -1.8 for IGFBP-3 (29) whilst in children with idiopathic short stature low IGF-I concentrations occur in around half of children (30) thus a degree of overlap is not unexpected. In addition, children with IGF-I concentrations in the upper half of the normal range may not have been selected for GH stimulation tests by their clinician. Other limitations of the previous study included the fact that GH assay and stimulation test were not standardized. GH assay has been standardized in our current study and while we did use two different pharmacological stimulation tests (glucagon and arginine) they are known to achieve similar peak GH levels (4). Our study is still limited by the small number of GHD subjects and further studies with larger cohorts are required to validate the Random Forest algorithm we have generated.

A wide range of genetic mutations are now known to cause GHD either alone or as part of a wider spectrum of hypopituitarism (31). An approach to identify DNA mutations known to be causative of GHD in these patients may allow such a diagnosis without a GH stimulation test or RNA based test such as the one described in this paper. As such it is likely that analysis of targeted gene panels will become part of the diagnostic pathway in the future but, given that a genetic etiology is not identified in the majority of GHD patients, DNA based analysis is unlikely to replace pharmacological stimulation tests in the near future. There are also a smaller number of patients with acquired childhood GHD where GHD is caused by tumors, radiotherapy, head injury etc. While a whole exome/genome approach may aid in diagnosing congenital GHD it will not be helpful in children with acquired GHD. These children will still require pharmacological stimulation tests or a replacement such as our gene expression-based test for the diagnosis of GHD.

Gene ontology analysis of the genes identified as having predictive power from Boruta highlighted mTOR signaling which is a known component of the insulin and IGF-I signal transduction pathways (32, 33) and is involved in cell growth, differentiation and metabolism. Inositol phosphate metabolic processes were also identified with inositol known to be involved in glucose metabolism and carcinogenesis (34) and myoinositol having been previously linked to poor intra-uterine growth (35). The two genes identified within the confirmed Boruta gene set from this study and also from the top 10 genes of highest predictive power in our previous study were *NRXN1* and *PTGDS*. Neurexin 1 (encoded by *NRXN1*) is a neuronal presynaptic cell adhesion molecule involved in synaptogenesis and vesicular neurotransmitter release (36). Deletions and loss of function mutations in *NRXN1* are associated with neurodevelopmental and psychiatric phenotypes (36). *NRXN1* has been linked to circadian rhythm in a genome-wide association study (37) with pathogenic copy number variants in *NRXN1* also linked to increased body mass index (38). *PTGDS* encodes an enzyme which catalyses the conversion of prostaglandin H2 to prostaglandin D2. The Ptgds^-/-^ mouse displays unilateral cryptorchidism (39) while low expression is linked to poor prognosis in endometrial cancer (40) and elevated levels linked to poor hair growth and androgenic alopecia in men (41).

In conclusion we have demonstrated a high degree of accuracy for diagnosis of childhood GHD utilizing a GE based test derived from a single blood sample expanding from our previous study to include short stature control subjects and the use of an RNA sequencing based approach. Further studies with greater numbers of patients are required to validate the random forest algorithm developed.

## Data availability statement

The datasets presented in this study can be found in online repositories. The names of the repository/repositories and accession number(s) can be found below: https://www.ncbi.nlm.nih.gov/geo/, GSE190502.

## Ethics statement

The studies involving human participants were reviewed and approved by the Bradford Leeds Research Ethics Committee (Reference 18/YH/0226 IRAS ID 231325). Written informed consent to participate in this study was provided by the participants’ legal guardian/next of kin.

## Author contributions

The study was conceptualized and designed by PM, AS, and PC. RNA sequence analysis was undertaken by TG and IW. PM drafted the manuscript. All authors contributed to the article and approved the submitted version.
