# Supplementary material for: Diagnosis of childhood and adolescent growth hormone deficiency using transcriptomic data
Source: Front Endocrinol (Lausanne). 2023 Feb 14;14:1026187. doi: 10.3389/fendo.2023.1026187 (PMC9973753; doi:10.3389/fendo.2023.1026187)
Supplement: Supplementary file 1 [file Table_1.docx]

Supplementary Table 1 – Individual Patient Data

| Gender | Age | Sex steroid priming | Peak GH (mcg/L) | GH stim test type | GHD | Other information |
| --- | --- | --- | --- | --- | --- | --- |
| M | 9.10 | Yes | 10.6 | Glucagon | No | Normal pituitary MRI, incidental finding arachnoid cyst |
| M | 12.20 | Yes | 10.4 | Glucagon | No | Normal pituitary MRI |
| F | 15.69 | No | 9.5 | Arginine | No |  |
| M | 6.61 | No | 8.7 | Arginine | No |  |
| M | 16.88 | No | 31 | Arginine | No | Wiedemann steiner syndrome |
| F | 13.13 | No | 9.1 | Arginine | No | Prader-Willi syndrome |
| F | 11.29 | Yes | 13 | Glucagon | No |  |
| M | 11.35 | Yes | 5 | Arginine | Yes | Small AP |
| M | 6.62 | No | 10 | Glucagon | No |  |
| M | 13.02 | No | 1 | Arginine | Yes | Small pit MR, arachnoid cyst |
| F | 14.27 | No | 2.6 | Arginine | Yes |  |
| F | 7.79 | No | 11.5 | Arginine | No |  |
| M | 13.88 | Yes | 10.1 | Glucagon | No | Normal MR pituitary |
| M | 9.25 | Yes | 15 | Arginine | No |  |
| F | 3.22 | No | 1.8 | Arginine | Yes |  |
| M | 3.76 | No | 4.6 | Arginine | Yes | MR pituitary normal |
| M | 5.95 | No | 3.8 | Arginine | Yes | Small AP |
| F | 12.59 | Yes | <0.1 | Glucagon | Yes | Small AP, thin stalk multiple sclerosis |
| F | 9.02 | No | 18 | Arginine | No |  |
| M | 3.62 | No | 12.4 | Arginine | No | Chromosomal abnormality 21q deletion |
| F | 8.25 | No | 7.4 | Arginine | No | Right middle cranial fossa arachnoid cyst, small AP |
| F | 5.50 | No | 25 | Arginine | No | Peroxisomal disorder |
| F | 4.17 | No | 12.1 | Arginine | No | Small AP |
| M | 3.49 | No | 2.4 | Arginine | Yes | NF1 |
